# Comparison of 2-hour versus 4-hour hemoadsorption combined with hemodialysis (HAHD): an in vitro and prospective clinical evaluation

**DOI:** 10.1080/0886022X.2025.2590229

**Published:** 2025-12-18

**Authors:** Wei Lu, Gengru Jiang

**Affiliations:** Department of Nephrology, Xinhua Hospital Affiliated to Shanghai Jiao Tong University School of Medicine, Shanghai, China

**Keywords:** Hemoadsorption, maintenance hemodialysis, uremic toxins, treatment duration, inflammatory cytokines, middle molecular toxins

## Abstract

Although hemoadsorption combined with hemodialysis (HAHD) is increasingly used in patients undergoing maintenance hemodialysis (MHD), the optimal treatment duration remains uncertain. This study compared the efficacy and safety of 2-hour versus 4-hour HAHD sessions, supported by *in vitro* kinetic modeling. An *in vitro* recirculation system was used to assess the adsorption kinetics of parathyroid hormone (PTH), β_2_‑microglobulin (β_2_‑MG), and tumor necrosis factor‑alpha (TNF‑α) over 4 h. Subsequently, 92 stable MHD patients were prospectively enrolled and assigned to receive a single HAHD session of either 2 h (*n* = 45) or 4 h (*n* = 47). Pre- and post-treatment blood samples were analyzed to determine the reduction ratios of inflammatory cytokines (IL-8 and TNF-α) and middle molecular toxins (PTH and β_2_-MG). *In vitro* results confirmed time-dependent adsorption, with 4-hour removal ratios for PTH (93.27%), β_2_-MG (93.36%), and TNF-α (50.54%) being significantly higher than those at 2 h (84.36%, 82.00%, and 27.56%, respectively; all *p* < 0.05). Clinically, the 4-hour group achieved significantly higher median reduction ratios for the inflammatory cytokines IL-8 (81.64% vs. 69.51%) and TNF-α (43.94% vs. 22.33%) and for the middle molecular toxins PTH (59.67% vs. 55.12%) and β_2_-MG (21.79% vs. 14.20%). Both durations were well tolerated, with no significant safety concerns. These findings indicate that prolonging HAHD to 4 h improves the clearance of inflammatory and middle molecular weight toxins without compromising safety and may offer clinical benefits for MHD patients with high inflammatory burdens.

## Introduction

Patients with end-stage renal disease (ESRD) have a high burden of uremic toxins, which are major contributors to the multi-systemic complications and high mortality rates observed in this population [1,2]. Beyond the accumulation of simple metabolic waste, ESRD is increasingly recognized as a state of chronic subclinical inflammation, where the retention of middle-to-large molecular weight solutes and protein-bound uremic toxins (PBUTs) perpetuates a vicious cycle of immune dysregulation, accelerated atherosclerosis, and protein-energy wasting [3,4]. Toxins such as β_2_-microglobulin (β_2_-MG), parathyroid hormone (PTH), and inflammatory cytokines are not only markers but also active participants in this inflammatory process [5,6].

To combat this, hemoadsorption (HA) has emerged as a valuable adjunctive therapy to conventional hemodialysis (HD) [7–10]. HA combined with HD (HAHD) offers a comprehensive approach to uremic toxin removal, and its use is supported by a growing body of evidence showing improvements in clinical symptoms and survival rates [11].

Despite the growing use of HAHD, optimal therapeutic parameters, particularly the duration of HA sessions, have not been well established and remain a subject of debate. Clinical practice guidelines often recommend a 2.0 to 2.5-h session, a duration rooted more in historical practice than kinetic evidence [12,13]. This limitation may prevent the full therapeutic potential of sorbents from being realized. Recent studies have begun to challenge this paradigm, suggesting that extending HA treatment to a full 4-h session can significantly enhance the clearance of various uremic toxins without compromising safety [14,15]. A multicenter prospective cohort study by Zhang et al. [14] explored the long-term safety of a 4-h HAHD protocol, providing important safety data for extending treatment duration. However, the efficacy analysis in that study was mainly based on a pre-post comparison using the patients’ own historical data. Consequently, a prospective study that directly compares the single-session efficacy of 2-h versus 4-h protocols between concurrent control groups is still lacking.

This study was designed to systematically investigate the impact of extending HA duration. We hypothesized that a 4-h HAHD session would be more effective than a standard 2-h session in removing key uremic toxins. We tested this hypothesis through a two-part study: first, an *in vitro* experiment to characterize the adsorption kinetics of a neutral macroporous resin (HA130, Jafron Biomedical Co., Ltd., Zhuhai, China); and second, a prospective clinical study to directly compare the efficacy and safety of 2-h versus 4-h HAHD in maintenance hemodialysis patients.

## Methods

### Part 1: *In vitro* adsorption study

#### Extracorporeal circuit and sorbent

An *in vitro* miniature extracorporeal circulation model was established to simulate clinical hemoadsorption. The circuit consisted of a peristaltic pump and custom-made tubing connected to a simulated HA cartridge. The cartridges were filled with 13 mL of HA130 neutral macroporous resin, representing a 1:10 scale model of the clinical HA130 device. This scaled-down model is a well-established and resource-efficient methodology for the preliminary evaluation of sorbent materials, as it allows for the accurate simulation of mass transfer kinetics. As described in other *in vitro* studies, the primary objective of this model is to elucidate the fundamental principles of adsorption dynamics and determine kinetic profiles, rather than to precisely replicate absolute clinical clearance ratios.

#### Plasma preparation and experimental protocol

Pooled plasma was obtained from stable MHD patients. For each experiment, 65 mL of pooled plasma was perfused through the circuit at 37 °C at a constant flow rate of 20 mL/min for 4 h. Plasma samples were collected at baseline (0h), 1 h, 2 h, 3 h, and 4 h. The experiments were performed in triplicate.

#### Measurements and calculations

Concentrations of PTH, β_2_-MG, and TNF-α were measured. The adsorption ratio (AR) at each time point was calculated using the formula:

AR (%)=(Cpre−Cpost)/(Cpre)×100%

where Cpre is the concentration at baseline (0h) and Cpost is the concentration at a given time point.

### Part 2: Prospective clinical study

#### Study design and patient population

A prospective, comparative, non-randomized study was conducted in 92 stable MHD patients who met the eligibility criteria and provided written informed consent. Based on their established therapeutic regimens and the clinical judgment of the attending physician, participants were assigned to one of two groups for a single study session – one underwent HAHD for 2 h (HAHD 2h group) and the other for 4 h (HAHD 4h group). Inclusion criteria comprised age ≥18 years, MHD vintage >3 months with a stable dialysis prescription (spKt/*V* ≥ 1.2), while exclusion criteria included active infection, malignancy, severe cardiovascular events within the past 8 weeks, and significant hematological disorders. The study protocol was approved by the Ethics Committee of Xinhua Hospital Affiliated to Shanghai Jiao Tong University School of Medicine (Approval No. XHEC-C-2017-042-2), and all participants provided written informed consent prior to enrollment. The study was conducted in accordance with the principles of the Declaration of Helsinki.

#### Treatment protocol

All HAHD sessions were conducted using a Fresenius 4008S machine, a low-flux polysulfone dialyzer (FX series), and an HA130 hemoadsorption cartridge. In the HAHD 2h group, patients received HAHD for 2 h. During these sessions, the HA130 cartridge was integrated into the hemodialysis circuit for the first 2 h. After 2 h, the cartridge was removed, and conventional HD continued for the remaining 2 h. In the HAHD 4h group, patients underwent HAHD for 4 h with the HA130 cartridge incorporated into the circuit throughout the entire session, following standard HD procedures without disconnection. For both groups, the blood flow rate was maintained at 200–250 mL/min, and the dialysate flow rate was set at 500 mL/min. Anticoagulation for all patients was achieved with an individualized regimen of unfractionated heparin. The protocol commenced with an initial weight-based intravenous loading dose of 62.5–125 U/kg (equivalent to 0.5–1.0 mg/kg) at the start of the session. Maintenance of anticoagulation was managed *via* intermittent intravenous boluses of 1250–2500 U/h (10–20 mg/h). The administration schedule for these maintenance doses differed by group: patients in the 2 h group received a single bolus at the end of the first hour, whereas patients in the 4 h group received boluses at the end of the first, second, and third hours.

#### Data collection and measurements

Blood samples were collected from the arterial line immediately before and after the respective treatment session. The samples were analyzed for concentrations of PTH, β_2_-MG, IL-8, and TNF-α. β_2_-MG levels were determined by immunoturbidimetry. The concentrations of PTH, IL-8, and TNF-α were measured using chemiluminescence assays. The reduction ratios (RR) for these toxins were calculated. Safety was evaluated by monitoring standard laboratory parameters and recording any adverse events.

### Statistical analysis

Statistical analysis was performed using SPSS version 22.0. Data were tested for normality. Comparisons between the two groups were performed using independent t-tests or Mann-Whitney U tests. A two-sided *p*-value of <0.05 was considered statistically significant.

## Results

### *In vitro* adsorption kinetics

**Figure 1.**
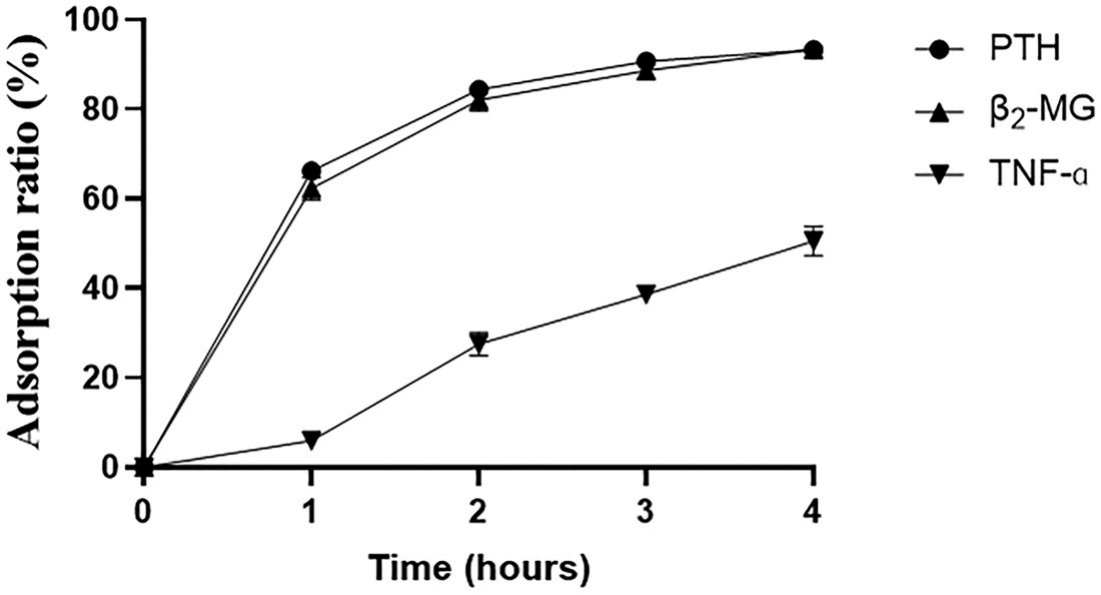
Time-course of uremic toxin adsorption *in vitro*. PTH: Parathyroid hormone; β_2_-MG: beta 2 microglobulin; TNF-α: tumor necrosis factor-alpha.

The *in vitro* experiment demonstrated that the adsorption of uremic toxins by the HA130 resin was time-dependent, as illustrated in Figure 1. The adsorption ratios for all measured toxins, detailed in Table 1, increased progressively over the 4-h perfusion period. At 2 h, the mean adsorption ratios were 84.36 ± 0.96% for PTH, 82.00 ± 2.38% for β_2_-MG, and 27.56 ± 2.57% for TNF-α. By 4 h, these ratios had increased significantly to 93.27 ± 0.16% for PTH, 93.36 ± 0.25% for β_2_-MG, and 50.54 ± 3.29% for TNF-α (*p* < 0.05 for all comparisons versus 2 h). This confirms that significant additional adsorption capacity is available beyond the standard 2-h treatment window.

**Table 1.** In vitro adsorption kinetics of uremic toxins over 4 h.

Time	PTH (pmol/L)	β₂-MG (mg/L)	TNF-α (pg/ml)	PTH AR (%)	β₂-MG AR (%)	TNF-α AR (%)
0h	33.61 ± 0.34	35.22 ± 0.37	14.53 ± 0.75	–	–	–
1h	11.37 ± 0.45	13.29 ± 1.01	13.67 ± 0.67	66.17 ± 1.65	62.27 ± 2.51	5.95 ± 1.49
2h	5.25 ± 0.28	6.34 ± 0.89	10.53 ± 0.61	84.36 ± 0.96	82.00 ± 2.38	27.56 ± 2.57
3h	3.12 ± 0.15	4.02 ± 0.27	8.91 ± 0.35	90.70 ± 0.49	88.59 ± 0.68	38.64 ± 1.43
4h	2.26 ± 0.04	2.34 ± 0.10	7.18 ± 0.33	93.27 ± 0.16	93.36 ± 0.25	50.54 ± 3.29

Data are presented as mean ± SD. PTH: Parathyroid hormone; β₂-MG: beta 2 microglobulin; TNF-α: tumor necrosis factor-alpha; AR: Adsorption Ratio.

### Clinical study results

#### Patient characteristics

A total of 92 patients were enrolled in the clinical study, with 45 allocated to the HAHD 2h group and 47 to the HAHD 4h group. The baseline demographic and clinical characteristics for both groups are presented in Table 2. Overall, the baseline data were similar between the two groups, with the exception of dialysis vintage, which was significantly longer in the 4h group (*p* < 0.05).

**Table 2.** Baseline characteristics of clinical study patients.

Parameter	HAHD 2h group (*n* = 45)	HAHD 4h group (*n* = 47)
Age (years)	64.0 (57.5, 69.0)	59.0 (51.0, 64.0)
Male n (%)	32 (71.1)	34 (72.3)
Dialysis vintage (months)	37.0 (26.5, 66.5)	68.0 (34.0, 128.0)
Hb (g/L)	111.20 ± 13.37	111.75 ± 14.41
WBC (×10⁹/L)	5.69 ± 1.43	6.09 ± 1.62
PLT (×10⁹/L)	182.07 ± 57.17	196.60 ± 58.84
CRP (mg/L)	3.00 (1.00, 6.50)	3.00 (1.00, 9.00)
Alb (g/L)	38.24 ± 3.91	39.77 ± 2.88
AST (U/L)	11.71 ± 5.28	10.53 ± 3.78
ALT (U/L)	10.38 ± 0.76	10.36 ± 4.13
Total bilirubin (μmol/L)	3.97 ± 1.68	4.40 ± 1.86
Direct bilirubin (μmol/L)	1.26 ± 0.60	1.42 ± 0.71
BUN (mmol/L)	25.96 ± 6.39	28.66 ± 6.34
SCr (μmol/L)	967.18 ± 208.84	1096.09 ± 259.86
UA (μmol/L)	435.47 ± 83.19	445.02 ± 88.50
Glucose (mmol/L)	10.29 ± 5.43	8.49 ± 3.44
Sodium (mmol/L)	135.65 ± 3.43	135.76 ± 2.64
Potassium (mmol/L)	4.71 ± 0.57	4.79 ± 0.59
Calcium (mmol/L)	2.11 ± 0.20	2.16 ± 0.23
Phosphorus (mmol/L)	2.17 ± 0.64	2.29 ± 0.58
Hcy (μmol/L)	44.37 ± 15.23	41.11 ± 12.36

Data presented as median (IQR) or mean ± SD. NS: Not Significant (*p* > 0.05). Abbreviations: Hb: Hemoglobin; WBC: white blood cell; PLT: blood platelet count; CRP, C-reactive protein; Alb: albumin; AST: aspartate aminotransferase; ALT: alanine aminotransferase; BUN: blood urea nitrogen; SCr: serum creatinine; UA: uric acid; Hcy: homocysteine.

#### Efficacy of toxin removal

Extending the treatment duration from 2 to 4 h showed a clear advantage in removing a broad range of uremic toxins. As detailed in Table 3, the 4-h HAHD group consistently demonstrated higher median reduction ratios compared to the 2-h group for all measured cytokines and middle molecular toxins.

**Table 3.** Comparison of uremic toxin reduction ratios between 2h and 4h HAHD groups.

Toxin RR (%)	HAHD 2h Group (*n* = 45)	HAHD 4h Group (*n* = 47)	Z/T value	*p*-value
IL-8	69.51 (31.10, 85.11)	81.64 (61.51, 95.90)	−2.839	0.005
TNF-α	22.33 (13.77, 50.51)	43.94 (28.57, 64.49)	−3.191	0.001
PTH	55.12 (27.01, 64.20)	59.67 (46.58, 72.89)	−2.566	0.010
β₂-MG	14.20 (5.00, 23.17)	21.79 (13.64, 44.40)	−3.050	0.002
BUN	63.91 ± 7.21	62.35 ± 7.18	1.038	0.302
SCr	59.93 ± 7.06	57.83 ± 6.96	1.432	0.156
UA	67.89 ± 7.65	67.14 ± 6.30	0.513	0.609
Phosphorus	56.68 ± 9.90	55.53 ± 7.60	0.626	0.533

Data are presented as median (IQR) and Mean ± SD. RR: Reduction ratio; HAHD: hemoadsorption combined with Hemodialysis; IL: interleukin; TNF-α: tumor necrosis factor-alpha; PTH: parathyroid hormone; β_2_-MG: beta 2 microglobulin; BUN: blood urea nitrogen; SCr: serum creatinine; UA: uric acid.

The improvement was particularly notable for inflammatory cytokines. The median RR for IL-8 was substantially higher in the 4-h group (81.64%) compared to the 2-h group (69.51%). Similarly, the RR for TNF-α was nearly doubled in the 4-h group (43.94%) versus the 2-h group (22.33%). For middle molecular toxins, the 4-h session also achieved higher RRs for both PTH (59.67% vs. 55.12%) and β_2_-MG (21.79% vs. 14.20%). There was no significant difference in the reduction ratios of BUN, SCr, uric acid, and serum phosphorus between the two groups (*p* > 0.05).

#### Safety assessment

Both treatment durations were well-tolerated, with no serious adverse events recorded in either group. Throughout the study, all scheduled treatments were successfully completed for their prescribed duration. There were no instances of premature session termination due to circuit or cartridge clotting in either the 2-h or the 4-h group. Post-treatment visual inspection of the circuit and cartridge revealed no significant differences in residual clotting between the two regimens. There were no statistically significant differences in the percentage changes of key safety markers, including white blood cell count, platelet count, albumin, and liver function enzymes (AST, ALT), between the 2-h and 4-h groups (Table 4).

**Table 4.** Comparison of percentage changes (%) in safety parameters.

Parameter percentage changes (%)	HAHD 2h group (*n* = 45)	HAHD 4h group (*n* = 47)	*Z* value	*p* value
WBC	−5.01 (−17.99, 7.03)	1.72 (−13.04, 10.00)	−1.125	0.261
PLT	5.51 (−3.67, 14.16)	−4.94 (−17.02, 4.26)	−1.032	0.302
Albumin	−13.62 (−19.08, −7.64)	−15.19 (−24.41, −10.46)	−1.222	0.222
AST	−30.77 (−48.08, −18.18)	−33.33 (−42.86, −20.00)	−0.195	0.845
ALT	−15.38 (−25.00, −6.94)	−16.67 (−27.27, −9.09)	−0.704	0.481

Data are presented as median (IQR). No significant differences were found between groups (*p* > 0.05 for all). WBC: white blood cell; PLT: blood platelet count; AST: aspartate aminotransferase; ALT: alanine aminotransferase.

## Discussion

This study provides both *in vitro* and clinical evidence that extending the duration of HAHD from the conventional 2 h to 4 h improves the removal of key inflammatory cytokines and middle molecular toxins without compromising safety. Our findings challenge the current standard practice and suggest that a longer treatment time is a simple and effective strategy to optimize blood purification for MHD patients.

The *in vitro* results clearly illustrate the foundational principle of time-dependent adsorption kinetics. As demonstrated in Figure 1, the adsorption kinetics for key toxins such as PTH and β2-MG show a steep and continuous uptake between the 2-h and 4-h time points. This clearly indicates that the sorbent is far from saturation at the conventional 2-h mark, and its adsorptive capacity remains highly effective. This kinetic profile directly explains why the 4-h clinical regimen achieved significantly greater toxin clearance. The traditional 2-h protocol effectively halts a therapeutic process that still possesses substantial adsorptive potential. This is consistent with the understanding that modern, highly biocompatible sorbents can be used for extended periods to maximize solute removal [9,16]. This provides a strong mechanistic rationale for exploring longer treatment durations in the clinical setting to fully leverage the therapeutic potential of the adsorbent. While the absolute reduction ratios in the clinical setting were, as expected, lower than in the idealized *in vitro* model, this discrepancy is well-understood to result from *in vivo* complexities such as toxin rebound from tissue compartments, continuous endogenous generation, and protein-binding kinetics.

Our clinical data directly translate these *in vitro* observations into patient-level benefits. The consistently higher reduction ratios in the 4-h group across all measured large- and middle-molecular toxins demonstrate a clear dose-response relationship with time. While our study focuses on surrogate biomarkers, the clinical relevance of these markers is well-documented by recent evidence. Elevated β_2_-MG is not only a key pathogenic factor in dialysis-related amyloidosis but also remains a robust predictor of cardiovascular and all-cause mortality, even in the modern era of high-flux dialysis [17]. Similarly, dysregulation of PTH is a cornerstone of chronic kidney disease-mineral and bone disorder (CKD-MBD), a systemic disorder directly linked to vascular calcification, fractures, and increased mortality risk [18]. Furthermore, the pro-inflammatory cytokines IL-8 and TNF-α are central mediators in the chronic inflammatory state that drives atherosclerosis, protein-energy wasting, and is strongly associated with adverse cardiovascular outcomes and mortality in hemodialysis patients [3,4]. Therefore, the enhanced clearance of these molecules, as demonstrated in the 4-h group, represents a tangible biochemical improvement that is mechanistically linked to the pathophysiology of major long-term complications. This superior anti-inflammatory effect positions longer-duration HAHD as a key therapy for patients with a pro-inflammatory phenotype. This enhanced clearance may also contribute to what has been termed the “Inflammation Mitigation Hypothesis” (IMH), which posits that effective toxin removal can downregulate the body’s chronic inflammatory state over time [19].

Our findings directly challenge the conventional wisdom of limiting HA sessions to 2-2.5 h. This study, in line with emerging evidence, suggests that a fully integrated 4-h session is not only therapeutically superior but also operationally simpler. This is supported by recent international studies where HAHD sessions longer than the conventional 2 h demonstrated clear benefits. For instance, a study in Spain by Maduell et al. with HAHD sessions lasting nearly 5 h, showed that adding hemoadsorption to low-flux hemodialysis significantly improved the removal of medium-sized molecules [20]. Similarly, a study from Chile by Ramírez-Guerrero et al. found that a 3.5 to 4-h HAHD session effectively removed harmful advanced glycation end products (AGEs) [21]. Furthermore, research in Indonesia by Puspitasari et al. linked 4 to 5-h HAHD sessions to significant long-term improvements in clinical symptoms like pruritus and sleep quality, as well as a reduction in inflammatory markers [22]. In addition, a recent Chinese multicenter cohort study by Zhang et al. demonstrated that a 4-h HAHD protocol was well tolerated and significantly enhanced the clearance of protein-bound toxins and overall dialysis adequacy, without increasing clotting risk or albumin loss [14]. Collectively, these studies corroborate our conclusion that a longer treatment duration is an effective strategy to maximize the therapeutic potential of HAHD. Our work also validates a more flexible 2-to-4-h approach and supports a stratified, patient-centered model where a 4-h, high-efficacy regimen can be targeted to patients with high inflammatory or toxin burdens. Furthermore, from a practical and cost-effectiveness standpoint, a single 4-h session simplifies the clinical workflow, reducing nursing workload and the risks associated with mid-treatment procedural changes, thereby strengthening the value proposition of the extended protocol.

Crucially, the enhanced efficacy of the 4-h session did not come at the cost of safety. Our clinical data showed no significant differences in the change ratios of key hematological and biochemical markers between the two groups, and all sessions were completed without interruption due to circuit clotting. Importantly, the slight post-treatment increase in serum albumin concentration, observed in both groups, was clarified to be an expected consequence of hemoconcentration from ultrafiltration, not adsorptive loss. The lack of a significant difference between the groups in this regard confirms the safety of the extended duration in terms of albumin handling. The absence of adverse events and the stability of parameters like albumin, white blood cells, and platelets confirm that a longer session is well-tolerated. This safety profile is a testament to the improved biocompatibility of modern sorbent materials and supports the feasibility of integrating a 4-h HA cartridge into a standard HD session.

This study has some limitations. First, the clinical study was comparative but not randomized, which could introduce selection bias. This is suggested by the significantly longer dialysis vintage in the 4-h group, which likely reflects a tendency to prescribe longer treatments to patients with more complex conditions. While the superior clearance we observed in this challenging population could be seen as strengthening our findings, we acknowledge that only a rigorous randomized controlled trial (RCT) can eliminate this potential confounding bias. Second, our evaluation was confined to a single treatment session, as the study was designed to provide a mechanistic rationale for extending treatment duration rather than to assess long-term benefits. Consequently, we focused on surrogate biomarkers like β_2_-MG, IL-8, and TNF-α. While these markers are well-established predictors of clinical outcomes, this study serves as a foundational precursor to our larger, completed RCT (NCT03227770) which provides definitive evidence on long-term, patient-centered outcomes. Furthermore, the statistical power may have been insufficient to declare all observed trends as statistically significant.

## Conclusion

In conclusion, this study demonstrates that extending the duration of HAHD from 2 to 4 h is a safe and more effective strategy for removing both inflammatory cytokines and middle molecular toxins. The *in vitro* data confirm that significant adsorbent capacity remains after 2 h, and the clinical data show that this can be translated into improved toxin clearance in patients. Our findings provide a strong rationale for reconsidering current clinical guidelines and for adopting longer-duration HAHD therapy, especially for patients with a high inflammatory or middle-molecule burden.

## Data Availability

The data underlying this article will be shared on reasonable request to the corresponding author.
